# Variations in the Microcystin Content of Different Fish Species Collected from a Eutrophic Lake

**DOI:** 10.3390/toxins5050992

**Published:** 2013-05-15

**Authors:** Justine R. Schmidt, Mylynda Shaskus, John F. Estenik, Carl Oesch, Roman Khidekel, Gregory L. Boyer

**Affiliations:** 1 Department of Chemistry, State University of New York, College of Environmental Science and Forestry, Syracuse, NY 13210, USA; E-Mail: glboyer@esf.edu; 2 Ohio EPA, Division of Surface Water, Lazarus Government Center, P.O. Box 1049, 50 West Town Street, Suite 700, Columbus, OH 43216, USA; E-Mails: Mylynda.Shaskus@epa.state.ohio.us (M.S.); john.estenik@epa.state.oh.us (J.F.E.); 3 Ohio EPA, Division of Environmental Services (Laboratory), 8955 East Main Street, Reynoldsburg, OH 43068, USA; E-Mail: carl.oesch@epa.ohio.gov (C.O.); roman.khidekel@epa.ohio.gov (R.K.)

**Keywords:** LC-MS/MS, microcystin-LR, accumulation, fish, cyanobacteria, toxin

## Abstract

Microcystins produced from cyanobacteria can accumulate in fish tissues. Liquid chromatography coupled with tandem quadrupole mass spectrometry (LC-MS/MS) is an attractive alternative to immunoassays for the determination of low concentrations of microcystins in tissues. Fish taken from Grand Lake St. Marys, a eutrophic lake in Ohio, USA, were analyzed for microcystin-LR in their fillets using LC-MS/MS. Of 129 fish tested for microcystins, only black crappie (*Pomoxis nigromaculatus*) and common carp (*Cyprinus carpio*) tested positive for microcystin-LR. Less than 10% of *Pomoxis* and 7% of *Cyprinus* samples contained measurable levels of microcystin-LR. Statistical analysis yielded a p-value of 0.07 between *Pomoxis* and the pooled results of the other four fish species. However, this comparison was complicated by the large difference in sample size between species. Further sampling in Grand Lake St. Marys for microcystin-LR would help determine if microcystin-LR exposure occurs through foodweb transfer.

## 1. Introduction

Cyanobacteria (blue-green algae) can proliferate in marine and freshwater bodies with high nutrient loads [1,2]. Selected species of cyanobacteria also produce toxic secondary metabolites. These toxic metabolites are classified into four categories: hepatotoxins, dermatoxins, irritant toxins, and cytotoxins, depending on their mode of action [3,4,5]. Of these, hepatotoxic microcystins are the most common and can usually be found in over half of the freshwater bodies where cyanobacteria are present [6]. The risk to human and ecosystem health due to the potentially increasing occurrence of these toxins is of concern [2,7].

Microcystin-LR is a monocyclic peptide hepatotoxin produced by cyanobacteria and is the most common variant of the larger family of related toxins collectively called microcystins [8]. These toxins impact liver tissue in organisms and cause cell damage and death through inhibition of protein phosphatases [9]. Microcystins enter terrestrial organisms predominantly through ingestion of water contaminated by a toxic cyanobacterial bloom. In aquatic organisms, the toxin can enter the organism by being absorbed through the gills when they filter contaminated water, or through the diet [8,10]. 

Microcystin-LR prevents protein phosphatase from functioning through noncompetitive inhibition by its 3-amino-9-methoxy-2,6,8-trimethyl-10-phenyl-4,6-decadienoic acid (ADDA) group. However, microcystins also form covalent linkages to a cysteine in the enzyme protein phosphatase though a Michael addition to the dihydroalanine residue [11]. Microcystins can be biotransformed into less toxic products, such as the glutathione conjugate [12,13]. These conjugates prevent covalent binding of the toxin to the protein phosphatase and promote its elimination from the body through the urine and feces [14,15,16] or bile [17,18,19].

Microcystins can accumulate in the tissues of organisms. Bivalves [15,20,21], snails [22,23], shrimp [22,23,24], and frogs [23] have all tested positive for microcystins after exposure to cyanobacterial blooms. In planktivorous organisms, microcystins accumulate in tissues through ingestion of toxic *Microcystis* in the diet [25,26,27,28], or via foodweb transfer [24,27]. More than 80% of the non-covalently bound microcystin in the zooplankton *Bosmina* fed to the sunfish *Lepomis gibbosus* was directly transferred to the sunfish, indicating that free or conjugated microcystin-LR can travel efficiently up the aquatic foodweb [27]. A summary of microcystin accumulation in several fish species is provided in Table 1. 

**Table 1:** Summary of microcystins found in fish tissue arranged by species. The range of microcystin detected, whether fresh weight (FW) or dry weight (DW) was used, and the extraction and analytical methods are included.

Fish species	Range of microcystin detected (µg/kg)	FW or DW	Extraction protocol	Analytical method	Author(s)	Year
Channel catfish (*Ictalurus punctatus*)	123.1–250.0	FW	Water:methanol:butanol (15:4:1) extraction, C18 cleanup	ELISA	[29]	2001
*Tilapia rendalli*	3.0–337.0	DW	100% methanol extraction	ELISA	[30]	2001
Goldfish (*Carassius auratus* L.)	50–300 (estimated from their Figure 1)	FW	100% methanol extraction	PPIA	[31]	2003
	500–1960 (estimated from their Figure 2)	DW	Water:methanol:butanol (15:4:1) extraction, C18 and Si cleanup	LC-PDA	[22]	2005
Yellow perch (*Perca flavescens*)	0.12–4.0	FW	75% methanol and acetic acid extraction	ELISA	[32]	2008
	0.5–7.0	DW	100% methanol extraction	ELISA	[33]	2011
Largemouth bass (*Micropterus salmoides*)	210.0–320.0	FW	Water:methanol:butanol (15:4:1) extraction, C18 cleanup	ELISA	[34]	2011
Nile tilapia *(Oreochromis niloticus)*	45-225 (estimated from their Figure 1b)	FW	Homogenization in methanol, hexane	LC-MS	[35]	2011
	0.8–63.4	DW	methanol extraction	ELISA	[33]	2011
Common carp (*Cyprinus carpio*)	46.3	DW	Water:methanol:butanol (15:4:1) extraction, C18 and Si cleanup	LC-PDA	[22]	2005
	3.3–19.0	FW	50% methanol, hexane	ELISA	[36]	2007
	2.85–138.7	FW	75% methanol, acetic acid	ELISA, LC-MS	[37]	2011
	50–470 (estimated from their Figure 4)	FW	100% methanol extraction	ELISA	[23]	2012
	3.5	FW	5% acetic acid, 0.01M EDTA extraction, charcoal	LC-MS/MS	Present study	2012
Black crappie *(Pomoxis nigromaculatus)*	399.0	FW	100% methanol and acidified water, cleanup with C18 cleanup	LC-MS/MS	[38]	2009
	1.5–1.9	DW	50% methanol extraction	ELISA	[33]	2011
	1.04–70.43	FW	5% acetic acid, 0.01M EDTA extraction, charcoal cleanup	LC-MS/MS	Present study	2012
White crappie (*Pomoxis annularis*)	270.0–320.0	FW	Water:methanol:butanol (15:4:1) extraction, C18 cleanup	ELISA	[34]	2011

Different species also vary in their sensitivity to microcystins [39]. Rainbow trout have withstood doses of microcystins which were lethal in mice [40,41] without mortalities. Hepatocyte hemorrhage was not observed in rainbow trout at concentrations lethal to mice, indicating that rainbow trout were more tolerant of microcystin exposure [42]. Rapid removal of microcystins from tissues was observed in several fish species, including common carp (*Cyprinus carpio*) and silver carp (*Hypoththalmichthys molitrix*) [36]. However, pre-exposure to microcystins may be required to induce this tolerance to microcystins [43,44,45]. *Daphnia galeata* hatched from resting eggs formed during periods of exposure to high microcystin concentrations were well-adapted to surviving subsequent microcystin exposure, while resting eggs formed during periods of exposure to low microcystin concentrations experienced a greater inhibition of growth [43]. The timing of when this exposure occurred is not always obvious. Microcystins have been detected in tissues of bivalves [20] and fish [30,46], even though no microcystins were detected in the water samples collected from these locations. Exposure to prior toxic blooms may be important in the organism’s ability to both resist intoxication and accumulate microcystins. 

Different analytical approaches have been used to test for microcystins in tissues (Table 1). Enzyme-linked immunosorbent assay (ELISA) is a useful tool for the rapid analysis of microcystins in water samples; however, its use with tissue extracts has limitations due to matrix effects that may lead to false positive results at low concentrations [47,48,49]. In response, many analytical laboratories have shifted to using liquid chromatography mass spectrometry (LC-MS(/MS)) for the analysis of microcystins in tissues [38,50]. Unlike the immunological assay, which provides an integrated response to all the microcystin variants in the sample, LC-MS(/MS) analysis differentiates between congeners of microcystins [50]. This provides important information on the congener distribution in the sample [46,50], but also makes it difficult to quantitate the total microcystin load in the tissues. Matrix interference can decrease the signal to noise ratio in LC-MS(/MS) analysis, increasing the detection limit and reducing the ability to detect microcystins at low concentrations. In response, tissue samples are routinely cleaned up prior to analysis using techniques such as solid phase extraction (SPE), or extraction with a non-polar solvent to remove lipids (Table 1). In this study, we used a novel charcoal-based solid phase extraction technique to prepare extracts for analysis. Norris *et al.* [51] used charcoal SPE to purify cylindrospermopsin from *Cylindrospermopsis raciborskii* culture media by eluting with 5% formic acid in methanol. Charcoal has been used to remove microcystins from drinking water [52,53,54]. Prior work on microcystin adsorption by charcoal did not consider removal of microcystins from charcoal once adsorbed; rather, the focus was the concentration of microcystins remaining in the solution after application of charcoal [54]. Few laboratories have used charcoal as a purification step prior to analysis. Here we use a charcoal purification step with liquid chromatography with multiple reaction monitoring of a quantitation transition and four individual confirmation transitions. 

## 2. Results and Discussion

The extraction protocol used here was a modification of the procedure of Zhang *et al.* [24] coupled with a cleanup procedure using charcoal SPE. The average recovery of microcystin-LR in water from charcoal SPE was 88.8% (range 63 to 105%, *n* = 20). Average recovery of microcystin-LR in fish muscle using charcoal SPE was 68.5% (range 54 to 106%, *n* = 23). Charcoal SPE effectively removed substances that interfered with the analysis of microcystin-LR by LC-MS/MS. The variation in retention time for microcystin-LR over a six month period was 1.05%, and the ion suppression of microcystin-LR by the fish tissue matrix was less than 3%.

The electrospray LC-MS/MS method used five individual transitions to provide a very high level of confidence in the identification of low levels of microcystin-LR in tissues. The five fragment ions of microcystin-LR selected for this analysis are shown in Table 2. The *m/z* 995→*m/z* 135 transition was derived from the loss of the ADDA group, and four individual confirmation transitions were from loss of an arginine residue and the Mdha moiety (Table 2). 

**Figure 1:**
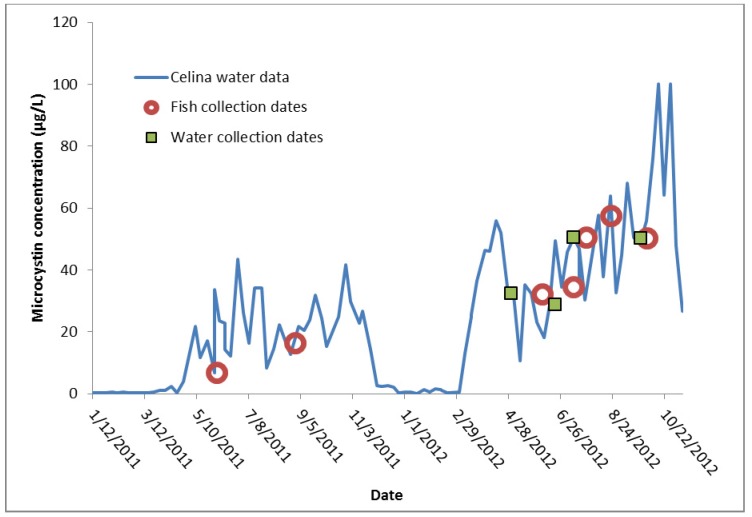
The dates of water and fish collection relative to the microcystin concentration (µg/L) in raw, unfiltered water collected from the Celina public water supply (PWS) water treatment plant over time. The City of Celina PWS is located on the northwestern shore of Grand Lake St. Marys, and the microcystin data is replotted from the public Ohio EPA database. Microcystin concentrations ranged from 0.2 µg/L to over 100 µg/L. Values over 100 µg/L are represented here as at 100 µg/L.

**Figure 2:**
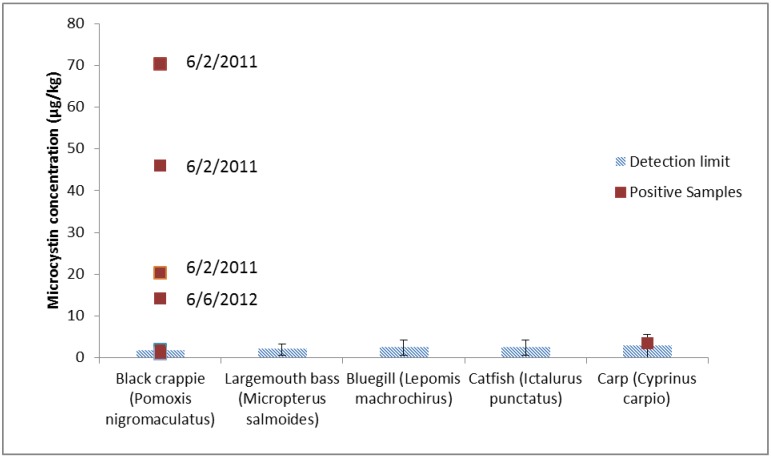
Microcystin-LR concentration in fish tissue by species. The detection limit for all samples is indicated by the striped bars. The collection date is given for positive *Pomoxis* samples greater than 10 µg/kg. In total, three positive *Pomoxis* samples were collected on 6/2/2011, three on 6/6/2012, and one on 7/25/2012. The single positive *Cyprinus* sample was collected on 8/30/2011.

Independent testing at a public water systems facility in the City of Celina (Celina PWS) on the shore of Grand Lake St. Marys reported that microcystin concentrations by ELISA ranged from 0.2 to over 100 µg/L in 2011 and 2012 [55] (Figure 1). Throughout 2011 and 2012, cyanobacteria dominated the total algal biovolume of the lake (range 6.72 × 10^9^ µm^3^/L on 8/31/2011 to 6.89 × 10^10^ µm^3^/L on 9/12/2012) [55]. Data on microcystin congeners for these samples is not available, but blooms at the City of Celina in 2010 contained predominantly microcystin-LR (Table 3). Blooms started in May and lasted through late November in both 2011 and 2012. Five species of fish were collected from different locations throughout the lake during the time period when cyanobacteria were present. Analysis of raw water samples collected within a few days of fish collection did not show measureable levels of microcystin-LR using LC-MS/MS (reporting limit < 0.01 µg/Liter; data not shown). Despite this absence of toxin in the water column, several fish tissues collected at the same time as water tested positive for microcystin-LR (Figure 2). There were a total of 10 positive samples in the quantitation ion, two of which could not be confirmed using the confirmation ions and were not assigned a numeric value. These two “trace” samples were both from *Pomoxis nigromaculatus*. The eight samples identified as positive in both the quantitation and confirmation ions include seven samples from *Pomoxis* and one sample from *Cyprinus carpio*. The microcystin-LR concentrations detected in *Pomoxis* ranged from 1.0 ± 1.4 µg/kg to 70 ± 5.0 µg/kg. Three positive *Pomoxis* samples were collected on 6/2/2011, three were collected on 6/6/2012, and one was collected on 7/25/2012. The concentration of microcystin-LR in the single *Cyprinus* was 3.5 ± 2.8 µg/kg, collected 8/30/2011. For the remaining 119 fish samples tested, microcystin-LR was not found above the quantitation limit (*ca.* 0.24 µg/kg FW) (Figure 2). There was no obvious relationship between the concentration in the fish and the concentration in the water samples collected from the same time and location as the fish.

**Table 2:** Instrument detection limit, method detection limit, and LC-MS/MS parameters for each transition of microcystin-LR.

Transition (*m/z*)	Instrument detection limit (µg on column)	Method detection limit (µg/kg)	Cone Voltage (V)	Collision Energy (V)
995→107	0.03	0.09	85	90
995→112	0.05	0.15	85	95
995→135	0.02	0.05	85	90
995→155	0.06	0.18	85	85
995→213	0.08	0.24	85	87

**Table 3:** Microcystin concentrations and congeners in algae samples collected at the City of Celina throughout 2010. Microcystin-LR was the dominant microcystin congener for all samples.

Date of collection	Microcystin concentration (µg/g dry weight)	Congeners
6/17/2010	132	100% microcystin-LR
7/19/2010	537	55% microcystin-LR
		28% microcystin-LW
		9% microcystin-YR
		8% microcystin-RR
8/11/2010	46	100% microcystin-LR
8/24/2010	20	100% microcystin-LR

A comparison of *Pomoxis* to all other species (*n* = 69 and 60, respectively) yielded a *p*-value of 0.07. *Pomoxis* and *Cyprinus* showed no differences (*p* = 1.0) with the different number of positive results in the two species, most likely due to the large difference in number of fish of each species collected (69 and 15 for *Pomoxis* and *Cyprinus*, respectively). Attempts to separate fish species on the basis of potential feeding behavior also did not show any significant differences, in part due to the small sample size and in part due to the complex feeding behaviors of many of these fish species. 

Grand Lake St. Marys is a large inland lake (*ca.* 5,500 ha.). Much of the watershed surrounding the lake is agricultural or used to raise livestock [56,57,58,59]. Anthropogenic nutrients from these operations can lead to increased concentrations of phosphorus and ammonium in the lake [56], which encourages the growth of algae. In recent years, the lake has experienced several large, toxic algal blooms that have been detrimental to wildlife, tourism, and business in the region [59] (Figure 1). In response, the Celina PWS uses granulated activated carbon filters, ozonation and chlorination to remove microcystins from water. This procedure was very effective at removal of microcystins from the raw feed water, with concentrations of microcystins in treated water below detection limit of ELISA in 2011 and 2012 [55]. Of 129 total fish collected, only 8 (*ca*. 7%) tested positive for microcystin-LR using a sensitive LC-MS/MS method. Seven out of the eight total positive samples were *Pomoxis nigromaculatus*, and the measured concentrations of toxins in these positive samples were usually tenfold greater than the method detection limit. Like *Micropterus* and *Lepomis*, *Pomoxis* eats from a variety of sources, such as zooplankton, small fish, and eggs [60]. However, concentrations in tissues were well above the concentration of microcystin-LR in the water column collected near the same time and location as the fish. Throughout 2011 and 2012, the algal community of Grand Lake St. Marys was dominated by *Planktothrix agardhii*. This cyanobacterium can produce toxins in European lakes [61], and the *mcy*A gene sequence coding for microcystin production in *P. agardhii* has been found in Lake Erie [62].

Microcystin blooms can be highly localized [63,64] and it is likely these fish were exposed to high concentrations of microcystins outside the site where they were captured. The most consistent monitoring of microcystin concentrations of Grand Lake St. Marys occurs at the City of Celina PWS, where microcystin concentrations peaked at greater than 100 µg/L during 2012. Thus, there was ample opportunity for the fish to accumulate microcystins from a separate toxic bloom prior to their capture by electroshocking. At this time, it is impossible to distinguish if this exposure occurred at a separate location in the lake, or if the exposure occurred at a separate time after which the bloom dissipated prior to collection of the fish samples. 

Differential accumulation of microcystins in fish tissues of different species has been noted by others (Table 1). However, a comparison across studies is difficult due to the different methods used for analysis. Poste *et al.* [33] sampled fish of several species from nine lakes across two continents and detected microcystins in fish at all sites using ELISA. The Nebraska Department of Environmental Quality also used ELISA to detect microcystins in *Micropterus salmoides* [34], unlike this study where all *Micropterus* samples were below the detection limit using LC-MS/MS. Microcystins were detected in white crappie (*Pomoxis annularis*) at levels from 270 to 320 µg/kg using ELISA [34] and in *Pomoxis nigromaculatus* at over 300 µg/kg using LC-MS/MS [38]. These results are in reasonable agreement with the results reported here for *Pomoxis nigromaculatus* (up to 70 µg/kg). This suggests that fish of the genus *Pomoxis* may more readily accumulate microcystins in tissues than other genera. We did not observe positive results for catfish (*Ictularus*) tissues, which have been reported to contain microcystins using ELISA [29]. 

The large difference between sample sizes for different fish species as well as the large number of non-detects made it difficult to compare *Pomoxis* to the pooled results of *Micropterus*, *Ictularus*, *Lepomis*, and *Cyprinus*. Further studies with an increased number of samples from each species would be necessary to see if fish accumulate microcystins from the diet, or through contaminated water passing over the gills. 

These results also may also reflect the differences in analytical methods used, and suggest that care must be taken when reporting low levels of microcystin concentration in fish based solely on the results of ELISA. Moreno *et al.* [48] suggest that matrix effects inhibit ELISA at microcystin concentrations less than 5.9 µg/kg dry weight, making this a useful tool for tissue analysis only when microcystin-LR concentrations are high. Most of our samples would have fallen below that threshold, indicating that ELISA may not be suitable for quantitation of microcystin-LR in these fish tissues.

**Figure 3:**
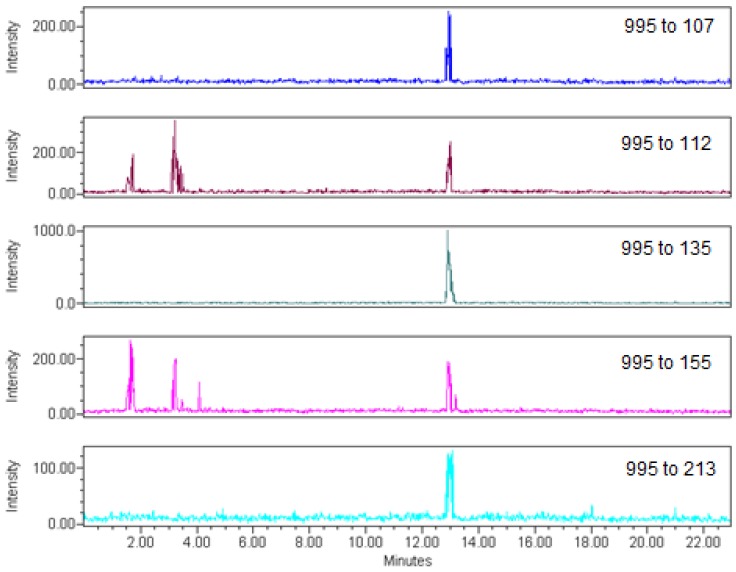
LC-MS/MS chromatograms of a positive *Pomoxis* sample from Grand Lake St. Marys, collected 6/6/2012. The third chromatogram shows the *m/z* 995 to 135 transition, which was used to quantitate microcystin-LR in the samples. The remaining four chromatograms show transitions used for the confirmation of microcystin-LR in a sample.

The positive samples in our study were unambiguously identified for microcystin-LR in tissues using five transitions in LC-MS/MS (Figure 3). Other studies have used LC-MS to detect microcystins in tissues [31,35,37]. However, this technique can overestimate the microcystins present in a sample [65]. Full scan analysis using LC-MS often lacks the necessary sensitivity for tissue analysis, and microcystins do not suitably fragment using collision-induced fragmentation under positive electrospray conditions to allow for the use of multiple selective ion monitoring (SIM). Environmental Protection Agency methods for SIM in the United States generally require at least three independent ions for quantitation and confirmation [66]. The limited fragmentation that is generally observed with microcystins under positive electrospray ionization makes this metric difficult to implement using a single quadrupole mass spectrometer. While it may be possible to induce additional fragmentation through collision-induced dissociation [67], most analysts have switched to tandem quadrupole mass spectrometry, where fragmentation is induced in the collision cell. Use of a single transition of the molecular ion to a fragment ion to both identify and quantify the desired analyte is often practiced. This generally is not sufficient in tissue samples, where there may be a large number of interfering ions due to the tissue matrix. Here, we use five individual transitions to identify microcystin-LR in these samples, thus providing a high degree of confidence in the identification of positive samples and limiting any false positives. 

To protect human health, the World Health Organization (WHO) recommended the tolerable daily intake (TDI) for microcystins over the lifetime of an individual be 0.04 µg microcystin-LR equivalents per kg of body weight per day [68,69,70]. Ibelings and Chorus [70] used the seasonal WHO guideline value of 0.4 µg/kg body weight/day to estimate a seasonal (daily exposure for several weeks during the cyanobacterial season) tolerable intake for microcystin-LR in food. Converting this laboratory value to the amount of fish that can be safely consumed is dependent upon several factors, including body weight of the affected individual, amount of contaminated fish consumed per day, the duration and mode of exposure, and several uncertainty factors. Using a safety factor of 100, a body weight of 10 kg for a child and 75 kg for an adult, and 100 g of contaminated fish being consumed per day, Ibelings and Chorus derived a seasonal value of 300 µg/kg of food for adults and 40 µg/kg of food for children. Levels for lifetime exposure to microcystin-LR using similar assumptions and safety factors would be tenfold lower. Additional safety factors may need to be included for children [41,71]. Using these numbers, none of the fish tissues tested in this study would have exceeded the seasonal value for adults. However, two out of the eight positive samples would have exceeded the seasonal value for children or the lifetime value for adults. Mulvenna *et al.* [71] assign an acceptable daily limit for microcystins in fish of 39 µg/kg of fish for adults (aged 17 and above) and 24 µg/kg of fish for children (aged 2–16). This was based on the No Observed Adverse Effect Level (NOAEL) of 40 µg/kg/day derived from studies in mice [68], and uncertainty factors of 10 (interspecies variation), 10 (intraspecies variation), and 2 (limited data on microcystins in freshwater organisms). A factor of 319 g of fish consumed/day for children and 377 g/day for adults was used to generate the guideline values. Average body weights of adults and children were 74 and 38 kg, respectively [68]. Using the guidelines of Mulvenna *et al.*, two of the eight positive samples in our study would have been above the limit for children, and only one positive sample would be above the limit for adults. 

The current study suggests that *Pomoxis nigromaculatus* is more at risk for microcystin contamination than other species of fish. However, this conclusion is complicated by several factors. Most of the *Pomoxis* samples were negative for microcystin-LR, while only *ca.* 10% were positive. This could be due to population differences in the lake, with *Pomoxis nigromaculatus* in one location in the lake exposed to more toxic algal blooms than other populations. These differences could also arise from differences in the diet between populations. Little information is available on the ecosystem and foodweb of Grand Lake St. Marys. Data on the zooplankton population, sediment load of microcystins, and diet of fish in the lake could be used to determine if *Pomoxis* developed high concentrations of microcystin-LR in their tissues from their diet. Exposure could have occurred through foodweb transfer using the zooplankton as vectors, but this remains unknown without zooplankton toxicity data. The sample size for *Pomoxis* was considerably larger than that of the other species of fish in this study. This makes it difficult to draw any conclusions regarding microcystin accumulation of other fish species. Additional sampling with larger sample sizes for all species tested would help determine the statistical difference in microcystin accumulation and consumption risk between fish species.

This study was designed to measure only free microcystin-LR in tissues, as this was the dominant congener in the lake in 2010. Metabolic products of microcystins such as the microcystin conjugates using the glutathione metabolic pathway may also contribute to the overall microcystin content of the organism. Microcystins also form covalent linkages with the protein phosphatase enzyme. Protease degradation of that enzyme-microcystin complex should release peptides that have similar toxicity to the GSH conjugate [72]. It is estimated from the oxidation of the ADDA moiety in tissues that as much as 60%–90% of total microcystins in tissues are covalently-bound [73,74,75] and may be of interest when discussing toxicity. However, the toxicity of these covalent complexes is much less than that of free microcystin-LR [76,77,78,79]. Studies on the release of microcystins from these conjugate forms to free microcystins would need to be conducted to estimate their contribution to the total microcystin content of an organism.

## 3. Experimental Section

Grand Lake St. Marys (GPS coordinates 40.542733, -84.464822) is the largest inland lake in the State of Ohio, USA. Fish were collected during the summer season using an electrofishing boat during the daytime. Fish were killed before being wrapped in aluminum foil and frozen using dry ice. Samples remained frozen until being ground using a meat grinder (large fish size samples) or blender (small fish size samples) [80]. The meat grinder, blender, and other tissue preparation hardware were thoroughly cleaned with hot detergent water, distilled water, and pesticides quality acetone before and after each fish sample grinding. Fish samples were chopped into 1 cm^3^ or smaller chunks using a meat cleaver for the blender and larger fish chunks for the meat grinder. Before grinding, fish samples were mixed with approximately one-half tissue volume of dry ice. Samples were ground to a powder, with dry ice added as necessary to keep the samples frozen. Samples were passed through the meat grinder twice to ensure they were pulverized completely. Samples were split after processing and one aliquot was wrapped in aluminum foil for organic analysis. The second aliquot was put into a Whirl-pak, frozen, packed with dry ice, and shipped to the State University of New York-College of Environmental Science and Forestry for toxin analysis. A total of 129 fish were collected, including 69 black crappie (*Pomoxis nigromaculatus*) and 15 each of bluegill (*Lepomis macrochirus)*, channel catfish (*Ictalurus punctatus*), common carp (*Cyprinus carpio*), and largemouth bass (*Micropterus salmoides*).

Tissue samples were extracted using a modified procedure of Zhang *et al.* [24]. Briefly, 400 mg tissue (fresh weight) was extracted with 5 mL of 5% acetic acid, 0.01 M EDTA solution with ultrasound (20 Watts) for 2 minutes at room temperature. Extracts were centrifuged (24,000 g) and applied to an activated charcoal solid phase extraction cartridge (SPE) (500 mg, part number 12252201) preconditioned with 3 mL of 5% formic acid in methanol and 3 mL Nanopure water. The SPE cartridge was eluted with 25 mL of methanol containing 5% formic acid and the elute was taken to dryness *in vacuo* using a rotary evaporator. The sample was reconstituted in 1 mL of 80% methanol and analyzed with LC-MS/MS. Water samples were extracted in 1% acetic acid, 50% methanol solution with ultrasound for 1 minute, with 20 second pulses, on ice [81]. Extracts were then centrifuged (12061 g) for 10 minutes prior to syringe-filtering before analysis by LC-MS/MS.

Tissue and water extracts were analyzed for microcystin-LR using a Waters 2695 Separations Module coupled to a Waters TQD tandem mass spectrometer. Column conditions consisted of an ACE^®^ C18 column (MacMod Analytical, 2.1 × 150 mm, 3 µm, 200 µL/min) with a 23 minute gradient of 30% acetonitrile in water containing 0.02% trifluoroacetic acid (TFA) to 100% acetonitrile containing 0.02% TFA. The electrospray LC-MS/MS method used five positive ion transitions for microcystin-LR; *m/z* 995→*m/z* 135 was used for quantitation, and *m/z* 995→*m/z* 107, *m/z* 995→*m/z* 112, *m/z* 995→*m/z* 155, and *m/z* 995→*m/z* 213 were used to confirm the identity of microcystin-LR in positive samples (Figure 3). Instrument detection limits, method detection limits, and LC-MS/MS conditions are summarized in Table 2.

A Fisher’s Exact Test was carried out on all fish samples using Minitab (Minitab Incorporated) software. Statistical analysis compared the *Pomoxis nigromaculatus* against *Cyprinus* alone and against the pooled results of the four remaining species. Samples without detectable levels of microcystin-LR were included in the statistical treatment as non-values. This was due to the large difference in sample size between *Pomoxis* and the other species. 

## 4. Conclusions

There may be a difference in microcystin-LR content in the tissues of fish based upon species. *Pomoxis nigromaculatus* and *Cyprinus carpio* were the only two out of five total species which tested positive for microcystin-LR. However, only *ca.* 10% of *Pomoxis* samples and 6.6% of *Cyprinus* samples were positive. A p-value of 0.07 was obtained when comparing *Pomoxis* to the pooled results of the other fish species, suggesting that this species may be more sensitive to the accumulation of microcystins that other species. This exposure did not coincide with cyanobacterial toxins in the water at the time of collection. Additional sampling of fish and toxin content of zooplankton would help answer the question of how fish are exposed to microcystin-LR.
